# PPAR-Mediated Toxicology and Applied Pharmacology

**DOI:** 10.3390/cells9020352

**Published:** 2020-02-03

**Authors:** Yue Xi, Yunhui Zhang, Sirui Zhu, Yuping Luo, Pengfei Xu, Zhiying Huang

**Affiliations:** 1 School of Pharmaceutical Sciences, Sun Yat-sen University, Guangzhou 510006, China; 2 Center for Pharmacogenetics and Department of Pharmaceutical Sciences, University of Pittsburgh, Pittsburgh, PA 15213, USA

**Keywords:** PPARs, toxicology, pharmacology, ligand

## Abstract

Peroxisome proliferator-activated receptors (PPARs), members of the nuclear hormone receptor family, attract wide attention as promising therapeutic targets for the treatment of multiple diseases, and their target selective ligands were also intensively developed for pharmacological agents such as the approved drugs fibrates and thiazolidinediones (TZDs). Despite their potent pharmacological activities, PPARs are reported to be involved in agent- and pollutant-induced multiple organ toxicity or protective effects against toxicity. A better understanding of the protective and the detrimental role of PPARs will help to preserve efficacy of the PPAR modulators but diminish adverse effects. The present review summarizes and critiques current findings related to PPAR-mediated types of toxicity and protective effects against toxicity for a systematic understanding of PPARs in toxicology and applied pharmacology.

## 1. Introduction

Peroxisome proliferator-activated receptors (PPARs), a group of nuclear hormone receptors, are composed of three isoforms which were identified as PPARα, PPARγ, and PPARβ. Each is encoded by distinct genes and has different targeting ligands, tissue distribution, and biological activities. PPAR family proteins, like other nuclear receptors, have three main functional segments, activation function 1 (AF1) and the conserved DNA-binding domain (DBD), the hinge region, and the ligand-binding domain (LBD) and AF2. The variable N-terminal regulatory AF1 domain binds co-regulators and the conserved DBD, which can bind to the peroxisome proliferator response elements (PPREs). The mobile hinge region links DBD and the conserved LBD in the middle. LBD and the variable C-terminal AF2 domain form a large ligand binding pocket [1,2]. Due to the large LBD pocket, PPARs have the capacity to bind various compounds, including endogenous or synthetic ligands and xenobiotic chemicals. In pharmacology, the ligands of PPARs are classified into full agonists, partial agonists, neutral antagonists, and inverse agonists. Recently, we summarized the 84 types of PPAR synthetic ligands for the treatment of various diseases in current clinical drug applications [3]. The LBD contains a C-terminal AF2 motif that is a ligand-dependent activation region [4]. Under physiological conditions, PPARs bind with co-repressors and form heterodimers with retinoid X receptor (RXR) [5]. In response to ligand activation, the protein conformation is changed and stabilized, which leads to dissociation of co-repressors and the recruitment of transcription co-activators and DNA-binding cofactors. This complex regulates transcription of target genes by binding specific DNA sequences, called peroxisome proliferator response elements (PPREs), on promoter regions of target genes [6,7].

PPAR activated genes play critical roles in fatty acid transportation and catabolism, glucose metabolism, adipogenesis, thermogenesis, cholesterol transportation and biosynthesis, and anti-inflammatory response [4,8]. Because of their broad-spectrum biological activities, PPARs arouse much attention, and they are studied intensively. Accumulated studies show that activation of PPARs has unique pharmacological effects on cardiovascular function, neurodegeneration, inflammation, cancer, fertility, and reproduction, and it is well established for managing dyslipidemia, diabetes, insulin resistance, and metabolic syndrome, which stimulates researchers to persistently develop more new drugs targeting PPARs [9,10]. Some PPAR agonists are approved as clinical agents such as thiazolidinediones, fibrates, and glitazars, for the treatment of diabetes, dyslipidemia, and diabetes-associated complications, respectively.

Despite the multiple biological activities of PPARs, several studies and clinical cases indicated that PPARs mediate various adverse effects of drugs, especially PPAR ligands or xenobiotic chemical-induced toxicity in different systems. Thiazolidinediones (TZDs), one class of PPARγ agonists, can cause fluid retention, heart failure, and hepatotoxicity [11,12]. Glitazones, another form of PPARγ ligand, were reported to cause peripheral edema, congestive heart failure, and body weight gain. Gemfibrozil, as a valuable agent to coronary heart disease, was shown to induce tumorigenesis, muscle weakness, and liver hypertrophy [13]. The detailed information and adverse reactions or toxicity of the 18 approved clinical agents that target PPARs are summarized in Table 1. Because of the high prevalence of tumorigenesis in PPAR activation by synthetic compounds, the Food and Drug Administration (FDA) requires that any PPAR agonists undergo a two-year rodent carcinogenicity study before being tested in clinical trials [13]. Moreover, PPARs were shown to be involved in pollutant-induced toxicity in the cardiovascular system, liver, reproductive and developmental system, gastrointestinal tract, muscle, and nervous system.

Based on the above information, this review is focused on the reports of PPAR activation-mediated toxicity and protective effects to date, aiming to provide an overview of studies evaluating the toxic role of PPARs in various systems and the molecular mechanisms of PPAR-elicited toxicity.

## 2. PPARs in Cardiotoxicity

Considering the high expression level of PPARs in cardiac muscles and their strong implication in metabolic disorders and endocrine disruption, interference with PPARs can affect metabolic homeostasis and development of the cardiovascular system.

Cardiac edemas and the impairment of cardiac development were observed in marine medaka larvae fish exposed to perfluorooctane sulfonate (PFOS) by interfering PPARα and PPARβ [14,15]. Perfluorooctanoic acid (PFOA) exposure-induced right-ventricular wall thinning elevation in chicken embryos is also likely due to PPARα [16]. After exposure to di-ethyl-hexylphthalate (DEHP), changes in the metabolic profile via the PPARα pathway can be detected in rat cardiomyocytes [17]. Triclocarban (TCC) is a high-performance broad-spectrum fungicide, which can induce cardiac metabolic alterations in mice by suppression of PPARα messenger RNA (mRNA) expression and other enzymes involved in energy and lipid metabolism. A further study found TCC directly interacted with the active site of PPARα in both mice and human tissues [18]. Exposure to airborne particulate matter is positively correlated with cardiorespiratory mortality [19]. Some studies showed that heart abnormal energy metabolism caused by seasonal ambient fine particles (PM_2.5_) was related to PPARα-regulated fatty-acid and glucose transporters. Unmanaged heart abnormal energy metabolism eventually leads to cardiac damage and heart failure [20]. Considerable research suggested that PPARs play pivotal roles in myocardial energy dysfunction. Energy substrate utilization showed a marked shift from fatty acid to glucose and lactate and cardiac hypertrophy in PPARα^−/−^ hearts [21]. PPARα-null hearts with decreased contractile and metabolic remodeling were rescued by enhancing myocardial glucose transportation and utilization [21].

Furthermore, PPARγ inhibits cardiac growth and embryonic gene expression and decreases nuclear factor kappa B (NF-κB) activity in mice [22]. Cardiomyocyte-specific PPARγ knockout mice were more susceptible to cardiac hypertrophy with systolic cardiac function [22]. CKD-501, a new selective PPARγ agonist, induced heart toxicity in db/db mice by PPARγ-dependent mechanism [23]. Rosiglitazone leads to cardiac hypertrophy partially independent of cardiomyocyte PPARγ [22]. Another study indicated that rosiglitazone caused oxidative stress-induced mitochondrial dysfunction via PPARγ-independent pathways in mouse hearts [24]. Anna et al. reported that atorvastatin ameliorated cardiac hypertrophy by improving the protein expression of PPARα and PPARβ, which regulated the gene expression involved in fatty acid metabolism and avoided NF-κB activation by reducing the protein–protein interaction between PPARs and p65 [25]. Moreover, atorvastatin reduced the paraquat-induced cardiotoxicity via the PPARγ pathway [26]. Hesperidin, a flavanone glycoside and a known PPARγ ligand, improved cardiac hypertrophy by improving cardiac hemodynamics, as well as inhibiting oxidative stress and apoptosis through increasing PPARγ expression [27]. Piperine, a phenolic component of black pepper, attenuated cardiac fibrosis via PPARγ activation and the inhibition of protein kinase B (AKT) / glycogen synthase kinase 3 β (GSK3β) [28]. Interestingly, the regulation of PPARγ by pioglitazone suppressed cardiac hypertrophy as indicated by decreased heart/body weight ratio, wall thickness, and myocyte diameter [29], but the effect of pioglitazone on limiting myocardial infarct size was a PPARγ-independent event [30]. Epoxyeicosatrienoic acids (EET), a primary arachidonic acid metabolite, blocked tumor necrosis factor α (TNFα)-induced cardiotoxicity by reducing inflammation via upregulation of PPARγ expression [31].

Some dual PPARα/γ agonists such as tesaglitazar display an increased risk of cardiovascular events. Treatment with tesaglitazar in mice caused cardiac dysfunction associated with low mitochondrial abundance [32]. In addition, tesaglitazar increased acetylation of proliferator-activated receptor gamma coactivator 1α (PGC1α) and decreased the expression of sirtuin 1 (SIRT1), which was associated with competition between PPARα and PPARγ. LY510929, another dual PPARα/γ agonist, was shown to cause left-ventricular hypertrophy in rats [33]. However, aleglitazar inhibited hyperglycemia-induced cardiomyocyte apoptosis by activation of both PPARα and PPARγ [34,35]. Activation of PPARβ signaling mediated docosahexaenoic acid (DHA), and its metabolites elicited cytotoxicity in H9c2 cells via the de novo formation of ceramide [36]. Doxorubicin (DOX) caused a remarkable decrease in cardiac dP/dT and cardiac output by inhibition of PPARβ expression in rats [37].

**Figure 1:**
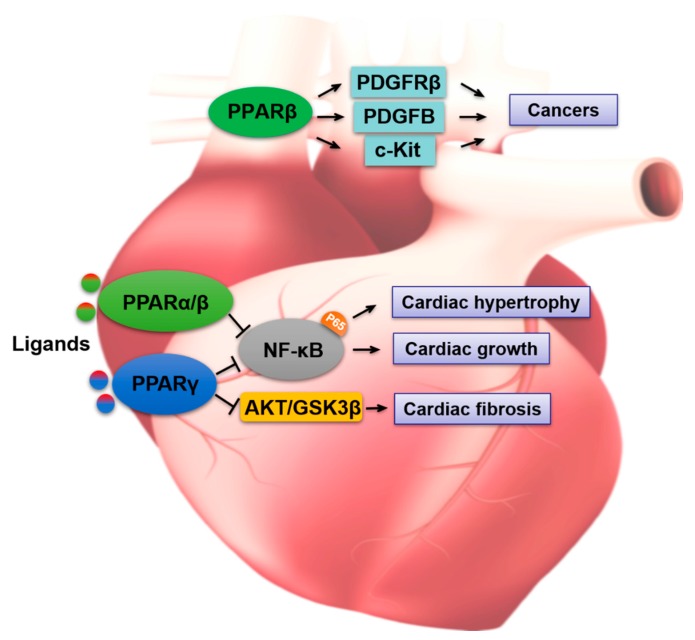
The regulation of PPARs in cardiotoxicity.

PPARβ plays an important role in angiogenesis and cancers. Activation of PPARβ in blood vessels promotes tumor vascularization and the progression of different cancer cell types through direct activation of platelet-derived growth factor receptor beta (PDGFRβ), platelet-derived growth factor subunit B (PDGFB), and the c-Kit [38]. Figure 1 summarizes regulation of PPARs in cardiotoxicity.

## 3. PPARs in Hepatotoxicity

In the liver, PPARs play indispensable roles in fatty-acid and glucose metabolism, and they supply energy to peripheral tissues. Numerous studies reported that xenobiotic chemicals and environmental contaminants disrupted the normal liver homeostasis by activating PPAR subtypes that are highly expressed in hepatocytes, especially PPARα. Indeed, PPARα was recognized as a target for pollutants, which could interact with the similar nuclear receptors and subsequently induce metabolic disorders.

Phthalates, common plasticizers in nearly all plastic consumer goods, are defined as PPAR modulators [6]. Accumulative studies showed that phthalates activated PPARα and other lipid-activated nuclear receptors in the liver, which induced metabolic disruption and endocrine disorders. The exposure concentration of phthalate metabolites such as DEHP and mono (2-ethylhexyl) phthalate (MEHP) positively correlated with insulin resistance and abdominal obesity in American male adults [39,40,41]. Di-*n*-butyl-di-(4-chlorobenzohydroxamato) tin (DBDCT), an organotin with high antitumor activity, was also demonstrated to induce notable toxicity in rat liver tissue via the PPAR signaling pathway [42]. DBDCT treatment aroused acute and focal necrosis and Kupffer cell hyperplasia in rat liver. The decreased expression levels of cluster of differentiation 36 (CD36), fatty acid binding protein 4 (FABP4), enoyl-CoA hydratase and 3-hydroxyacyl CoA dehydrogenase (EHHADH), acetyl-CoA acyltransferase 1 (ACAA1), phosphoenolpyruvate carboxykinase (PEPCK), PPARα, and PPARγ in DBDCT-treated liver tissue were indicated by proteomics. Furthermore, the toxic effect was alleviated by PPARγ blocking agent T0070907 [42,43]. Additionally, organotins, the major components of agricultural fungicides and pesticides, were documented to exert similar functions as PPARγ and PPARβ ligands, which promote weight gain and increase fat storage by target gene induction in liver [44]. For example, tributyltin chloride (TBT) enhanced adipogenesis and adipocyte differentiation by directly stimulating downstream transcription of PPARγ in liver and adipose tissues. In mouse models, uterus exposure to TBT disrupted hepatic architecture and caused liver steatosis by increasing lipid accumulation and adipocyte maturation [40,45]. Thus, PPARs play an important role in contaminant-induced toxicity.

The hepatotoxicity of PPARα ligands was rarely documented. Few PPARα ligands are proven to be hepatotoxicants. Fenofibrate exerts only a minimal increase of alanine aminotransferase and aspartate aminotransferase [6,46]. In contrast to hepatotoxicity mediated by PPARs, PPAR ligands also display some protective effect against hepatotoxicity. PPARα ligand activation was proven to prevent acute liver toxicity induced by alcohol, carbon tetrachloride (CCl_4_), acetaminophen, chloroform, thioacetamide, and bromobenzene due to the induction of fatty acid catabolism and anti-inflammatory properties [47,48,49]. PPARα agonists showed a reversal of fatty liver in mice even with continued ethanol consumption [50]. PPARγ agonist troglitazone and rosiglitazone are reported to induce mild liver toxicity in patients that might be PPARγ-independent because of the low expression level of PPARγ in the liver [12]. Despite the hepatotoxicity of PPARγ activation, PPARγ ligand treatment attenuated fibrogenesis by inhibiting the activation of hepatic stellate cells (HSCs) [51,52]. PPARγ ligands exhibited a suppressive effect on the expression of fibrogenic genes including collagen and α-smooth muscle actin. PPARβ activation by L-165041 enhanced the HSC proliferation and fibrogenic gene expression, and it exacerbated CCl_4_-induced liver fibrotic progression [53]. PPARα, PPARγ, and PPARβ display different roles in hepatotoxicity. Activation of PPARα prevents acute liver toxicity. Activation of PPARγ induces mild liver toxicity but attenuates liver fibrogenesis. Activation of PPARβ promotes the progression of liver fibrosis.

Numerous studies reported that hepatocarcinogenesis was the major toxicity induced by PPARα activation [54,55]. Unmanaged peroxisomal proliferation and hepatomegaly observed in fibrate-treated livers can ultimately lead to hepatocellular carcinoma [56]. The hepatocarcinogenesis by PPARα activation was fully investigated over 30 years. The main target of PPARα is the liver, which induces pleiotropic impacts such as hypertrophy and hyperplasia [57,58]. These unmanaged responses cause hepatocellular carcinomas in rodents. The mechanisms remain elucidated. Some studies propose that PPARα-mediated DNA replication, proliferation, and suppressed apoptosis result in PPARα agonist-induced hepatocarcinogenesis [59]. Actually, the effect of PPARα on hepatocarcinogenesis varies among different species. In human, an increased risk of liver cancer of fibrates is not yet reported. This might be due to no significant peroxisome proliferation induced by hypolipidemic agents [60] and less expression of PPARα in patient livers compared to rodent liver. Although humans show resistance to the adverse effect of PPARα-induced hepatocarcinogenesis, vigilance is still required to develop new agents.

## 4. PPARs in Gastrointestinal Toxicity

As indicated by emerging evidence, PPARs and their ligands also play an important role in the regulation of immune and inflammatory reactions in the gastrointestinal (GI) system.

In view of modulation of several target genes involved in metabolic processes and immune response in the GI tract, PPARs and their ligands became a research hotspot in gastroenterology [61]. Accumulative evidence showed that inflammatory bowel diseases (IBDs) and colon cancer (CC), two important GI diseases, are related to PPARs and their ligands [62,63]. PPAR agonists might serve as a new effective pharmacotherapy for IBDs and CC. PPARα mediated the anti-inflammatory effect of glucocorticoid (GC) in a chemical-induced colitis mouse model [64]. More recently, it was shown that PPARα activation diminished the therapeutic effects of rSj16 in dextran sulfate sodium (DSS)-induced colitis mice, indicating that the PPARα signaling pathway plays a crucial role in DSS-induced colitis progression [65].

With the high expression in GI tract mucosa, especially in the intestine and colon [66,67,68], PPARγ is closely related to GI injury and inflammatory response. The inflammatory reaction is the common pathological process of many GI diseases and trauma. Once the homeostasis of GI is disrupted by exogenous factors or endogenous metabolites and shifts to the pro-inflammatory state, the pro-inflammatory cytokines such as TNF-α, interleukin 1β (IL-1β), IL-6 are liberated by the hyperactive immune cells. Transcription factor NF-κB is one of the most important regulatory mechanisms of immune and inflammatory responses mediated by PPARs and their ligands in the GI tract. In colon, PPARγ downregulated NF-κB and mitogen-activated protein kinase (MAPK) signaling pathways, which subsequently inhibited the mucosal production of inflammatory cytokines [69]. Furthermore, in intestinal cells, activation of PPARγ resulted in decreased expression of intercellular adhesion molecule 1 (ICAM-1) and TNF-α [70], which are downstream targets of NF-kB [71]. Treatment with troglitazone attenuated colitis induced by intrarectal administration of 2,4,6-trinitrobenzene sulfonic acid (TNBS) [69]. PPARγ could function as an endogenous anti-inflammatory pathway in a murine model of intestinal ischemia–reperfusion (I/R) injury. Activation of PPARγ by its agonist BRL-49653 had a protective effect on intestinal acute I/R injury [70]. However, the protective activity of BRL-49653 was abolished in PPARγ-deficient mice. In the investigation of the prevention and treatment of radiation-induced intestinal damage, accumulating evidence supported that the administration of PPARγ agonists alleviated radiation-induced intestinal toxicity. PPARγ agonists were shown to reverse radiation-induced apoptosis and inflammation and to exert radio-protective effects on healthy bowel upon irradiation [72,73]. Further research of acute intestinal injury reported that PPARγ agonist rosiglitazone reduced the expression of the fibrotic marker transforming growth factor β (TGFβ) and phosphorylation of the p65 subunit of NF-kB triggered by pro-inflammatory cytokine TNF-α [72]. In ulcerative colitis research, promoting the nuclear localization of PPARγ weakened the activity of NF-κB signaling in both rectal tissues from dextran sulfate sodium (DSS)-induced mice and lipopolysaccharide (LPS)-stimulated macrophages [74]. PPARs inhibited the expression of macrophage-related inflammatory mediators and macrophage infiltration in the acute irradiation intestinal damage [75]. Compared with wild-type mice, PPARγ-deficient mice showed significantly severe damage after an I/R injury procedure, which indicated the anti-inflammatory and protective role of PPARγ in GI damage [70]. These studies indicated the role of PPARγ in suppression of NF-kB activation and inflammatory response in intestinal tissues.

Additionally, several reports indicated that PPARs and their ligands could lead to carcinogenesis by affecting the metabolism of glucose and lipids. Intestinal PPARα exhibited a protective effect against colon carcinogenesis by inhibiting methylation of P21 and P27 [76]. Human colorectal tumors also show lower levels of PPARα compared to normal tissue [76]. PPARγ synthetic activator rosiglitazone has a radio-sensitizing effect on human bowel cancer cells [72]. PPARγ was reported to be associated with colorectal cancer via insulin and inflammatory mechanisms [77,78]. On the other hand, PPARγ was shown to be expressed in human colonic mucosa and cancer. The ability of PPARγ activation to decrease cyclooxygenase-2 (COX-2) expression and induce apoptosis suggests that the PPARγ pathway might be a tumor suppressor in humans [79]. Another study reports that 8% of primary colorectal tumors harbor function-dead mutations in one allele of the *PPARγ* gene and emphasizes the potential role of this receptor as a therapeutic target for cancer or in designing a mouse colon cancer model [80]. The treatment of colon cancer by suppressing the methylation of PPARγ promoter and enhancing PPARγ expression is also underway, because the hyper-methylation of promoter regions can induce PPARγ gene silence. Moreover, the risk of radiation-induced intestinal toxicity in methylated patients was also increased compared with un-methylated patients [81]. Furthermore, PPARβ is induced in intestinal stem cells and progenitor cells in high-fat diet-treated mice and enhances stemness and tumorigenicity of intestine [82]. Arachidonic acid derivative prostaglandin E2 (PGE2), which is a biologically active lipid, increases cell survival and improves intestinal adenoma formation by indirectly activating PPARβ via the phosphatidylinositol-3 kinase (PI3K)/Akt signaling pathway [83]. The activation of PPARβ also upregulates COX-2, which is a key activator for colon cancer cells [59].

**Figure 2:**
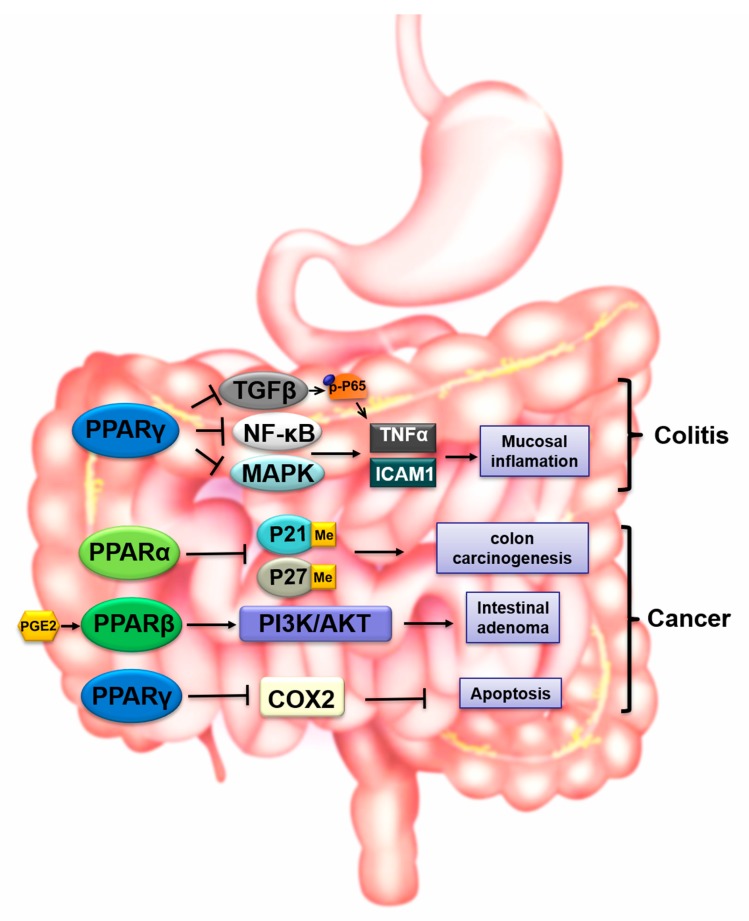
The regulation by PPARs in gastrointestinal toxicity.

We summarize the regulatory mechanism of PPARs in gastrointestinal toxicity in Figure 2. A better understanding of the role of PPARs in the GI system will help to develop novel pharmacotherapy against colon carcinogenesis and diminish intestinal toxicity.

## 5. PPARs in Reproductive and Developmental Toxicity

Three isoforms of PPARs were found in the reproductive system including the hypothalamus, pituitary, testis, ovary, uterus, adrenal, and mammary glands. Numerous studies showed that PPARs play a role in the normal reproductive and developmental functions, and abnormal regulation of PPARs by exposure to endogenous or exogenous compounds might lead to physiological dysfunction in reproductive system [84,85,86]. Thus, the research on reproductive and developmental disorders focuses on PPARs and their modulators.

Triptolide is a major active compound in Chinese herb *Tripterygium wilfordii* multiglycoside, and it is widely used for treatment of autoimmune diseases and nephrotic syndrome [87]. However, we previously reported that triptolide causes mitochondrial damage and dysregulates fatty-acid metabolism by upregulating expression and nuclear translocation of PPARα in mouse sertoli cells [88]. A metabolomics study revealed that triptolide caused impairment of spermatogenesis accompanied by abnormal lipid and energy metabolism in male mice through downregulation of PPARs [89]. Different concentrations and times of triptolide exposure led to the different behaviors of PPARs. These findings support that PPARs are key mediators in triptolide-induced reproduction toxicity.

Phthalates, which activate PPARs, have a remarkable effect on fertility rates, ovulation, development of the male reproductive tract, spermatogenesis, and teratogenesis [6]. Early exposure to phthalates influenced perinatal and postnatal cardiometabolic programming [90]. DEHP, a phthalate ester, is commonly used in industry as a plasticizer, which activates PPARα to regulate the expression of downstream target genes. DEHP treatment had no remarkable effect on body, liver, and ovary weight in female dams (F0) and offspring (F1) in either wild-type or PPARα-knockout mice. However, it suppressed the expression of ovarian estrogen receptor α, and the repression of ovarian estrogen receptor α expression by DEHP was lost in PPARα-knockout mice [91]. PPARα transcription is related to fertility impairment in female mice exposed to high doses of DEHP (500 mg/kg of body weight per day) [92]. Moreover, it was reported that MEHP, a principle active metabolite of DEHP, decreased the activity and production of aromatase, which converted testosterone to estradiol in ovarian granulosa cells by activating PPARα and PPARγ [93]. Benzo [a]pyrene (B [a]P) is a ubiquitous environmental contaminant, and the combination of B [a]P and DEHP induced ovotoxicity in female rats and suppressed sex hormone secretion via the PPAR-mediated signaling pathway [94].

Dehydroepiandrosterone (3c-hydroxy-5-androsten-17-one, DHEA) is a ligand of PPARα, and it also stimulates the production of PPARα. Some clinical studies showed that dietary supplementation of DHEA reversed the oocyte quality in mice and aged women [95,96]. Additionally, reduced DHEA and loss of function of PPARα result in the decreased follicle quality associated with the changes of fatty-acid metabolism, transport, and mitochondrial function. Perfluorooctanoic acid (PFOA), a synthetic perfluorinated compound (PFC) which is widely distributed, significantly inhibited mammary gland growth in mice through activation of PPARα, and this effect was reversed by supplementation with exogenous estrogen or progesterone [97]. Moreover, perfluorooctane sulfonate (PFOS) is a product of metabolic degradation of PFCs and has an estrogenic activity and endocrine-disruptive properties in the marine medaka embryos, partially through the regulation of PPARs.

Additionally, 15-deoxy-delta12,14-prostaglandin J2 (15dPGJ2), which is converted by arachidonic acid via successive dehydration and isomerization, acts as an endogenous ligand of PPARγ via direct covalent binding, and it plays a key role in lipid homeostasis [98,99]. Kurtz and colleagues found that 15dPGJ2 partially restored the mRNA expression of oxidizing enzymes including acyl-CoA 1 (ACO) and carnitine palmitoyltransferase 1 (CPT1) in the lungs of male fetuses from diabetic rats, but this effect was not observed in female fetuses [100]. Moreover, it was reported that 15dPGJ2 modulated lipid metabolism and nitric oxide production in diabetes-induced placental dysfunction partially through the PPAR pathway [101]. Trichloroethylene (TCE) reduced fertilizability of oocyte and its ability to bind sperm plasma membrane proteins in rats [102]. A systematic evaluation of TCE showed that TCE could cause cardiac defects in humans when the exposure is during a sensitive period of fetal development [103]. Tributyltin chloride (TBT) activates all three types of PPARs. TBT has effects on reproductive function and induces abnormal mammary gland fat accumulation by increasing PPARγ expression [104,105]. TZDs (e.g., pioglitazone, rosiglitazone, and troglitazone) activate PPARγ to regulate the transcription of genes responsible for glucose and lipid metabolism. TZDs clinically sensitize peripheral insulin in patients with type 2 diabetes by regulating glucose and lipid metabolism [106,107]. Oral administration of rosiglitazone 4 mg once a day for three months improves hyperandrogenemia, insulin resistance, lipidemia, C-reactive protein levels, ovarian volume, and follicle number in patients with polycystic ovary syndrome (PCOS) [108]. Rosiglitazone exhibited significant protective effects on metabolic, hormonal, and morphological features of PCOS. Significant changes were also observed in the isovaleryl carnitine levels and lipid oxidation rates after pioglitazone treatment [109]. Rosiglitazone significantly improved oocyte quality in diet-induced obesity (DIO) mice, indicating the positive effect of PPARγ on ovarian function [110]. Rosiglitazone affects steroidogenesis in porcine ovarian follicles by stimulating PPARγ [111,112]. In vivo experiments demonstrated that fenofibrate inhibited ovarian estrogen synthesis [113]. A review concluded that clofibrate and gemfibrozil caused atypical changes in maternal and fetal liver during pregnancy, but there was no direct evidence of developmental toxicity or teratogenicity of clofibrate and gemfibrozil [6]. Irbesartan (IRB) is one of the most widely used angiotensin type 1 (AT1) receptor blockers (ARBs) with PPARγ agonistic activity. Rats treated with IRB showed an increase in estradiol and follicle-stimulating hormone levels, which subsequently ameliorated ovarian dysfunction [114]. These studies indicate that activation of PPARγ signaling protects ovarian function.

Genistein (49,5,7-trihydroxyisoflavone, GEN), a kind of isoflavones derived from soybeans, was investigated for its antioxidant, anticancer, and anti-inflammatory activities [115]. It is a natural ligand of PPARs, and it can improve the development and metabolism of chick embryos through the activation of PPARs [116,117]. Prostacyclin (PGI2) activated its nuclear receptor PPARβ to accelerate blastocyst hatching in mice [118]. These studies suggest that the activation of PPARs is involved in toxicant-induced reproductive toxicity.

The anti-tumor effects of PPAR agonists were documented. Rosiglitazone and troglitazone, both PPARγ activators, showed inhibitory effects on pituitary adenoma cells in mice and human, and they were considered to be a new oral drug for the treatment of pituitary tumors [119]. Moreover, troglitazone treatment stabilized the prostate-specific antigen levels in patients with advanced prostate cancer clinically by upregulating E-cadherin and glutathione peroxidase 3 [120]. Rosiglitazone showed an inhibitory effect on proliferation of primary human prostate cancer cells [121]. However, the activation of PPARβ by selective agonist GW501516 was reported to stimulate proliferation of human breast and prostate cancer cells which are responsive to sexual hormones [122]. PPARβ activation by GW501516 increased cyclin-dependent kinase 2 (CDK2) and vascular endothelial growth factor α (VEGFα) expression, indicating the improved cell proliferation and angiogenesis. This study suggested the possibility of PPARβ antagonists in treating breast and prostate cancer.

## 6. Other Systemic Toxicity and Protective Effects Mediated by PPARs

Fibrates, PPARα synthetic ligands, were developed for treatment of hyperlipidemia in the clinic, such as fenofibrate, bezafibrate, ciprofibrate, and so on [123,124,125]. However, muscle weakness, muscle pain, and even rhabdomyolysis were observed during their application [6]. Different fibrates lead to different degrees of myopathy, and that might be due to different mechanisms. The underlying mechanism is still unclear. Some studies reported that PPARα activation in skeletal muscle transactivated the genes encoding muscle proteases, and the increased expression of skeletal muscle proteases led to severe myopathy [126,127]. The muscle toxicity might result from the blood concentration of the drug, because remarkably higher incidence occurs in patients with kidney failure or hypoalbuminemia [128]. Moreover, Motojma et al. proposed that the increase in pyruvate dehydrokinase isoenzyme4 (PDK4) and the decrease in serum triglyceride (TG) level mediated by PPARα in skeletal muscle caused the degradation of protein in muscle, ultimately resulting in myopathy and even rhabdomyolysis [129]. Due to the low incidence of rhabdomyolysis, no drug was withdrawn from the market because of the muscular toxicity.

In contrast to the adverse effect mediated by PPARs, PPARs also exert protective effects against nephrotoxicity and neuron injury.

Diabetic kidney disease is one complication of type 2 diabetes. PPARα and PPARγ are famous targets for treating diabetes, especially PPARγ. Increasing studies indicated that PPARs play important roles in kidney physiology and pathology. In most cases, PPARγ serves as a therapeutic target for treating nephrotoxicity. PPARγ-null mice showed spontaneous diabetic nephropathy. PPARγ knockout mice exhibited kidney hypertrophy accompanied by increased glucosuria, albuminuria, renal fibrosis, and mesangial expansion [130,131].

PPARs also play key roles in regulating brain self-repair. Central nervous system diseases, neuron injury, and cell death are closely related to neuroinflammation [132,133]. Lovastatin (LOV) can protect vulnerable oligodendrocytes in a mouse model of multiple sclerosis (MS) by inhibiting guanosine triphosphate (GTP)-binding proteins, small Rho GTPases, via a PPARα-dependent mechanism [134]. Healthy oligodendrocytes are essential for the synaptic survival of MS neurons. PPARα activation increases the seizure threshold and controls the seizure frequency [134]. The high expression of PPARα in the brain region also prevents nicotine-induced neuronal damage by regulating tyrosine kinases and phosphokinases in neuronal current. It decreases the frequency of seizures caused by the activation of nicotine receptors in vertebral neurons [135]. Animal model studies showed that fenofibrate prevented convulsions caused by dysregulation of neurotransmitters [136]. Substantia nigra has high-density microglia which show two polarization states, M1 and M2, which have pro-inflammatory or anti-inflammatory effects, respectively [132,137]. Therefore, inhibiting the activation of M1 microglia and promoting the activation of M2 microglia are beneficial to central system diseases. In the condition of inflammation, M1 microglia are activated and release pro-inflammatory factors and neurotoxic substances, such as cytokines, reactive oxygen species, prostaglandins, and complements, which aggravate inflammatory injury [138]. Recent studies showed that PPARs (mainly PPARγ) regulate microglia-mediated inflammation in Parkinson’s disease (PD) and other neurodegenerative diseases [138,139,140]. Pioglitazone, a PPARγ ligand, was shown to inhibit the activation and secretion of glial cells by activating PPARγ [141]. Pioglitazone also inhibits the degeneration of dopamine neurons, which induces inflammation and promotes neuron death [141]. Rosiglitazone has a protective effect on neurotoxin 1-methy-4-phenyl-1,2,3,4,6-tetrahydropyidine (MPTP)-induced PD mouse model via upregulation of M2 phenotypic-related anti-inflammatory factors and the downregulation of M1 phenotypic-related pro-inflammatory factors [142]. A recent study found that PPARα/γ dual agonist MHY908 protects dopamine neurons from MPTP-induced loss in PD mice by reducing neuroinflammation and microglia activation [141]. Moreover, L-165041, a PPARβ agonist, can inhibit the radiation-induced inflammation in microglia by inhibition of the NF-kB signaling pathway [143]. At present, Alzheimer’s disease is also considered to be a neuroinflammatory disease and is characterized by abnormal accumulation of β-amyloid (Aβ). Under the condition of Aβ accumulation, M1 microglia were activated, resulting in neuronal injury and apoptosis [144]. It was shown that adiponectin can activate M2 microglia and enhance the clearance of Aβ by activating the PPARγ signaling pathway.

## 7. Conclusions

**Figure 3:**
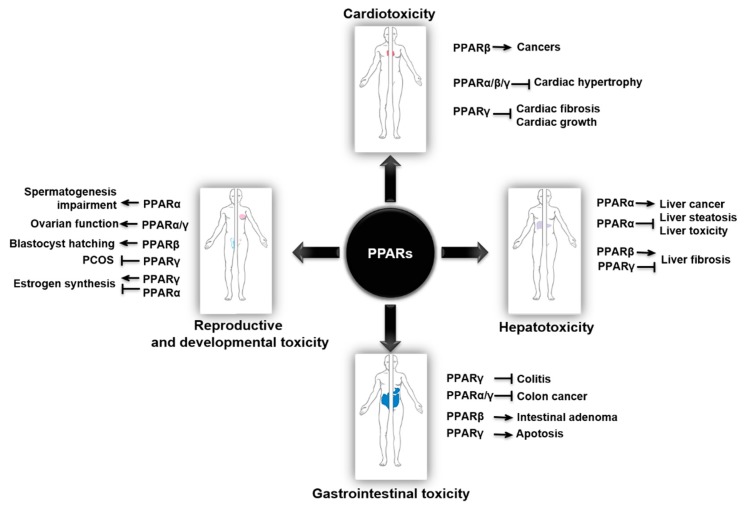
Concept map of the PPARs in various systemic toxicities.

Better understanding of the role of PPARs in toxicology and pharmacology and the underlying molecular basis is necessary for PPARs-related clinical drug discovery and development. Unfortunately, there are limited studies reviewing the integrated network of relationships in these aspects. Lots of PPAR ligands have beneficial effects on applied pharmacology, but they are also accompanied by various toxicities. Here, we mainly summarized the regulation of PPARs in toxicology and protection against toxicity in various systems, such as cardiotoxicity, hepatotoxicity, gastrointestinal toxicity, and reproductive and developmental toxicity (Figure 3). We hope that a comprehensive understanding of PPAR-mediated toxicology and applied pharmacology will contribute to the safety of PPAR-targeted therapies in the future.

## Figures and Tables

**Table 1:** The information and adverse reactions or toxicity of peroxisome proliferator-activated receptor (PPAR) targets related to 18 approved clinical drugs.

Generic Name (Brand Name)	Type of PPAR Agonist	Molecular Weight and Molecular Formula	Structure	Company	Indications	Adverse Reaction or Toxicity
Rosiglitazone maleate (Avandia)	PPARγ agonist	473.5 C_22_H_23_N_3_O_7_S	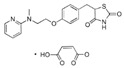	GlaxoSmithKline	Diabetes	Headache, cough, cold symptoms, and back pain
Pioglitazone hydrochloride (Actos)	PPARγ agonist	392.898 C_19_H_21_ClN_2_O_3_S	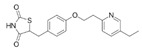	Takeda/Lilly	Diabetes	Cold or flu-like symptoms, headache, gradual weight gain, muscle pain, back pain, tooth problems, and mouth pain
Lobeglitazone sulfate (Duvie)	Dual PPARα/γ agonist	578.61 C_24_H_26_N_4_O_9_S_2_	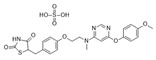	Chong Kun Dang	Diabetes	Edema and weight gain
Alogliptin benzoate/pioglitazone hydrochloride (Oseni)	Dipeptidyl peptidase IV inhibitor/PPARγ agonist	461.519(C_25_H_27_N_5_O_4_)/392.898 (C_19_H_21_ClN_2_O_3_S)	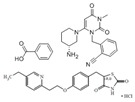	Takeda	Diabetes	Upper respiratory tract infection, bone fracture, headache, nasopharyngitis, and pharyngitis
Glimepiride/pioglitazone hydrochloride (Duetact)	Sulfonylurea receptor modulator/PPARγ agonist	490.62(C_24_H_34_N_4_O_5_S)/392.898 (C_19_H_21_ClN_2_O_3_S)	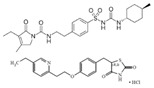	Takeda	Diabetes	Congestive heart failure, hypoglycemia, edema, fractures, and hemolytic anemia
Pioglitazone hydrochloride/metformin hydrochloride (Actoplus Met)	PPARγ agonist/ adenosine monophosphate-activated protein kinase (AMPK) activator	392.898 (C_19_H_21_ClN_2_O_3_S)/165.6(C_4_H_12_ClN_5_)	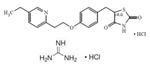	Takeda	Diabetes	Headache, nausea, vomiting, stomach upset, diarrhea, weakness, sore throat, muscle pain, weight gain, tooth problems, a metallic taste in the mouth, and sneezing, runny nose, cough, or other signs of a cold
Rosiglitazone maleate/metformin hydrochloride (Avandamet)	PPARγ agonist; AMPK activator	473.5(C_22_H_23_N_3_O_7_S)/165.6(C_4_H_12_ClN_5_)	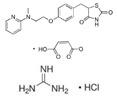	GlaxoSmithKline	Diabetes	Lactic acidosis, cardiac failure, adverse cardiovascular events, edema, weight gain, hepatic effects, macular edema, fractures, hematologic effects, and ovulation
Glimepiride/rosiglitazone maleate (Avandaryl)	Sulfonylurea receptor modulator/PPARγ agonist	490.62 (C_24_H_34_N_4_O_5_S)/473.5(C_22_H_23_N_3_O_7_S)	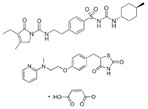	GlaxoSmithKline	Diabetes	Cardiac failure with rosiglitazone, major adverse cardiovascular events, hypoglycemia, edema, weight gain, hepatic effects, macular edema, fractures, hypersensitivity reactions, hematologic effects, hemolytic anemia, and increased risk of cardiovascular mortality for sulfonylurea drugs
Clofibrate (Atromid-S)	PPARα agonist	242.699 C_12_H_15_ClO_3_	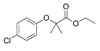	Pfizer	Hyperlipidemia Hypertriglyceridemia Hypercholesterolemia	Common: diarrhea, nausea Rare: abnormal heart rhythm, acute inflammation of the pancreas, anemia, angina, gallstones, kidney failure, and low levels of white blood cells
Fenofibrate (Antara)	PPARα agonist	360.834 C_20_H_21_ClO_4_	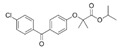	Abbvie	Hypercholesterolemia Hypertriglyceridemia	Common: abnormal liver tests including aspartate aminotransferase (AST) and alanine aminotransferase (ALT), and headache Rare: high blood pressure, dizziness, itching, nausea, upset stomach, constipation, diarrhea, urinary tract infections, muscle pain, kidney problems, and respiratory tract infections
Choline fenofibrate (Fenofibric Acid)	PPARα agonist	421.918 C_22_H_28_ClNO_5_	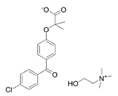	Abbvie	Hyperlipidemia	Diarrhea, dyspepsia, nasopharyngitis, sinusitis, upper respiratory tract infection, arthralgia, myalgia, pain in extremities, dizziness
Bezafibrate (Bezalip)	PPARα agonist	361.822 C_19_H_20_ClNO_4_	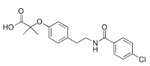	Roche Diagnostics	Hypertriglyceridemia hypercholesterolemia mixed hyperlipidemia	Stomach upset, stomach pain, gas, or nausea may occur in the first several days; itchy skin, redness, headache, and dizziness
Gemfibrozil (Lopid)	PPARα agonist	250.338 C_15_H_22_O_3_	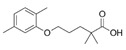	Pfizer	Hyperlipidemia Ischemic heart disorder	Stomach upset, stomach/abdominal pain, nausea, vomiting, diarrhea, constipation, rash, dizziness, headache, changes in the way things taste, muscle pain
Ciprofibrate (Lipanor)	PPARα agonist	289.152 C_13_H_14_Cl_2_O_3_	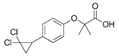	Sanofi-Aventis	Hyperlipidemia	Hair loss, balding, headache, balance problems, feeling dizzy, drowsiness or fatigue, feeling sick (nausea) or being sick (vomiting), diarrhea, indigestion or stomach pains, muscle pains
Pemafibrate (Parmodia)	PPARα agonist	490.556 C_28_H_30_N_2_O_6_	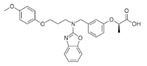	Kowa	Dyslipidemia	Cholelithiasis (upper abdominal pain, fever) and diabetes mellitus (dry mouth, excess intake of fluid, excessive urination, fatigue)
Pravastatin sodium/fenofibrate (Pravafenix)	3-hydroxy-3-methylglutaryl-CoA reductase (HMGCR) inhibitor/PPARα agonist	446.5(C_23_H_35_NaO_7_)/360.83(C_20_H_21_ClO_4_)	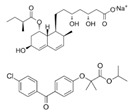	Laboratoires SMB	Mixed hyperlipidemia Coronary heart disease	Abdominal distension (bloating), abdominal pain (stomach ache), constipation, diarrhea, dry mouth, dyspepsia (heartburn), eructation (belching), flatulence (gas), nausea (feeling sick), abdominal discomfort, vomiting, and raised blood levels of liver enzymes
Fenofibrate/simvastatin (Cholib)	PPARα agonist/ HMGCR inhibitor	360.834 (C_20_H_21_ClO_4_)/418.57(C_25_H_38_O_5_)	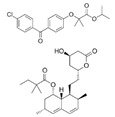	Mylan	Mixed hyperlipidemia	Raised blood creatinine levels, upper-respiratory-tract infection (colds), increased blood platelet counts, gastroenteritis (diarrhea and vomiting) and increased levels of alanine aminotransferase
Saroglitazar (Lipaglyn)	PPARα/γ agonist	439.57 C_25_H_29_NO_4_S	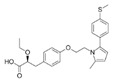	Zydus Cadila	Diabetic dyslipidemia	Asthenia, gastritis, chest discomfort, peripheral edema, dizziness, and tremors
